# Outcomes of acute kidney injury continuum in children

**DOI:** 10.1007/s40620-024-02097-1

**Published:** 2024-10-24

**Authors:** Flavia Chisavu, Mihai Gafencu, Ramona Stroescu, Lazar Chisavu, Adalbert Schiller

**Affiliations:** 1 University of Medicine and Pharmacy ‘Victor Babes’ from Timisoara, Eftimie Murgu, rue nr. 2, Timisoara, Romania; 2 ‘Louis Turcanu’ Emergency County Hospital for Children in Timisoara, Timisoara, Romania; 3 Centre for Molecular Research in Nephrology and Vascular Disease, Faculty of Medicine ‘Victor Babes’ from Timisoara, Timisoara, Romania

**Keywords:** Acute kidney disease, Acute kidney injury, Chronic kidney disease, Continuum

## Abstract

**Background:**

Acute kidney injury (AKI) is associated with high morbidity and mortality. The continuum of kidney damage after an AKI episode is poorly explored in the paediatric population.

**Methods:**

We performed a retrospective cohort study on 2346 children with AKI from a tertiary care hospital in Romania over a 9-year period. The main objective was to evaluate the impact of AKI duration on mortality and the risk of new-onset chronic kidney disease (CKD).

**Results:**

Out of 2346 AKI patients, transient AKI was present in 655 patients (27.9%), persistent AKI in 1009 children (43%) and acute kidney disease in 682 patients (29.1%). In contrast to transient AKI, children who developed acute kidney disease were younger, with a higher degree of anaemia, lower number of platelets, higher procalcitonin, higher LDH, higher GGT, higher urea and higher serum creatinine levels. The pre-renal cause of AKI was the leading cause regardless of AKI duration. As kidney injury progressed over time, there was an increasing incidence of the intrinsic causes of AKI (11.1% in transient AKI, 13.2% in persistent AKI and 22.6% in acute kidney disease). Acute kidney disease patients had the highest mortality rate (16.42%), followed by transient AKI (14.66%) and persistent AKI (9.81%). Overall mortality increased in the presence of renal microvascular alterations, acute tubular necrosis, lower haemoglobin, serum proteins and platelets, and higher procalcitonin levels.

**Conclusions:**

The continuum of AKI expressed as acute kidney disease resulted in an increased risk of new-onset CKD. CKD was influenced by the intrinsic cause of AKI and not by AKI severity.

**Graphical abstract:**

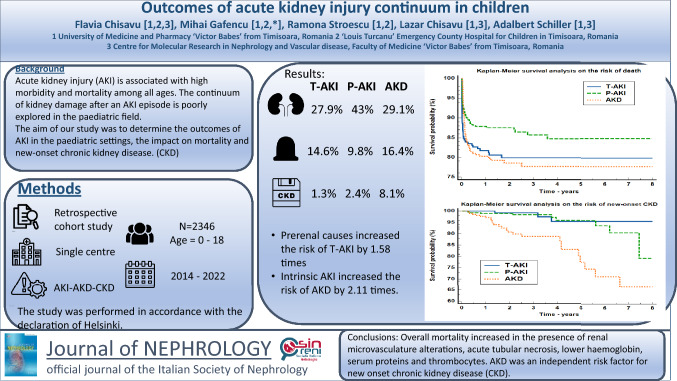

**Supplementary Information:**

The online version contains supplementary material available at 10.1007/s40620-024-02097-1.

## Introduction

Acute kidney injury (AKI) is a heterogeneous syndrome as multiple mechanisms contribute to kidney injury with varying intensity between and within patients. AKI severity and duration are reflections of the underlying disease spectrum. Mild AKI is defined by a transient decline in kidney function that usually involves rapid reversal of injury with no or minimal kidney function loss [1]. Yet, when rapid reversal of kidney injury does not occur within the first 48 h, AKI is defined as persistent AKI [2]. However, persistent kidney injury leads to kidney damage defined as acute kidney diseases or disorders. The definition of acute kidney disease has been proposed for persistent kidney injury lasting from 7 days to under three months [3, 4]. Although traditional approaches to acute alterations in kidney function with function-based stages and quasi-anatomic nomenclature are used as part of the standardised AKI definition and staging criteria, the concepts of transient and persistent AKI can add prognostic value to the actual definition.

Over the last decade, AKI and acute kidney disease have been acknowledged as independent risk factors for developing chronic kidney disease (CKD) and increased mortality in both adult and paediatric populations [5, 6]. Acute kidney disease may be superior to AKI in predicting the risk of CKD in children, but data are scarce [7]. CKD has been shown to be correlated to the severity of AKI, to the intrinsic cause of AKI, as well as to the severity of the underlying disease [8–12]. However, there is a lack of data regarding AKI duration and the risk of new-onset CKD or worsening CKD. The interplay between AKI–acute kidney disease–CKD in children remains to be established since only Patel reported the outcomes of acute kidney disease in children following non–kidney solid organ transplant [7]. On the other hand, few studies have reported acute kidney disease-associated mortality in the paediatric population [9, 13]. Given the lack of data regarding children, we performed a retrospective analysis on all children admitted to our hospital in order to report the outcomes of AKI duration and identify the associated risk factors in each timeframe. We also evaluated the continuum of AKI–acute kidney disease–CKD following the initial AKI episode. The main objective was to determine the relationship between AKI duration and severity and progression to CKD. In addition, we report the impact of subsequent AKI episodes in patients with and without CKD.

## Methods

### Ethical considerations

This study was conducted in accordance with the Declaration of Helsinki and approved by the Hospital’s Medical Ethics Committee (9485/20.06.2023). The requirement for informed consent was waived because of the retrospective observational nature of the study.

### Study design and participants

This large-scale, single–centre, retrospective observational cohort study of hospitalised children was performed between 1st July, 2014 and 31st December, 2022 at the ‘Louis Turcanu’ Emergency County Hospital for Children in Timisoara, Romania. A flow chart of the study is shown in the Supplemental figure. Patients aged 1 day to 18 years were screened based on the change in serum creatinine (SCr) through The Laboratory Information System and The Hospital Information System. Only the first hospitalisation was included if the patient had multiple admissions. After excluding 419 recurrent AKI episodes, the final cohort comprised 2346 patients with AKI.

### Data collection and definition

Baseline characteristics were obtained during the first admission (gender, environment, age, demographics). AKI was defined according to the 2012 KDIGO Clinical Practice Guideline [3] and 2021 Consensus Conference [6] as an increase in SCr by 26.5 µmol/l within 48 h or to more than 1.5 times baseline within 7 days. AKI staging was performed according to KDIGO SCr criteria in patients older than 1 month, while in neonates we used the modified KDIGO criteria [3, 14] as seen in Supplemental Table 1. Stage three AKI was considered in all patients with documented anuria for over 12 h.

Baseline creatinine was defined as the lowest serum creatinine in the 7 days prior to AKI diagnosis, or the minimum inpatient serum creatinine value for patients who met the criteria for community-acquired AKI in patients over 1 month old. In neonates, we applied the modified neonatal KDIGO criteria where the baseline SCr was the lowest value prior to the AKI episode.

Transient AKI was defined as patients having an AKI episode lasting 48 h.

Persistent AKI was defined as patients having an AKI episode longer than 48 h and less than 7 days [4].

Acute kidney disease was defined according to the 2017 ADQI consensus statement [4] in the eighth day of persistent AKI based on the SCr level.

Chronic kidney disease was defined according to the KDIGO CKD guideline as abnormalities of kidney structure or function (as evidenced by damage markers) lasting longer than three months, with health implications or GFR < 60 ml/min/1.73 m^2^ for more than three months with or without damage markers [15]. New-onset CKD was considered in patients who developed CKD after at least 3 months from the initial AKI episode.

All biological parameters were obtained on the first day of AKI. Follow-up was performed from the first day of AKI until July 2023, death or the last admission. During follow-up, we evaluated the risk of mortality stratified on AKI duration. The risk of new-onset CKD after an AKI episode was assessed in patients with more than 3 months of follow-up, after excluding patients with pre-existing CKD. In addition, we evaluated the risk of recurrent AKI in patients with CKD.

### Statistical analysis

Data are presented as average ± standard deviation (SD) for continuous variables and percentage for categorical variables. For continuous variables, we used ANOVA (the comparison between the three groups: transient AKI, persistent AKI and acute kidney disease). Categorical variables were analysed with the Chi–square test. The mortality risk was analysed using multivariate Cox proportional hazards models (HR). Odds ratio (OR) and 95% confidence interval (95%CI) were calculated. Kaplan Meier survival analysis was performed for the risk of death and CKD development. In order to assess the independent factors predicting the risk of death in our cohort, we employed a backward multivariable logistic regression model (in the entire cohort and in subgroups stratified by AKI duration). In this study, a P–value of 0.05 was considered the threshold for statistical significance. Data were analysed using MedCalc Statistical Software version 22.009.

## Results

Out of 142,762 hospital admissions, 2765 episodes of AKI were identified. The overall incidence of AKI was 1.64% after excluding recurrent AKI. The final cohort included 2346 patients with AKI.. Transient AKI was present in 655 patients (27.9%), persistent AKI (P–AKI) in 1009 children (43%), and 682 patients (29.1%) progressed to acute kidney disease. The baseline characteristics are presented in Table 1.

**Table 1:** Baseline characteristics of the entire cohort

	Transient AKI (1)	Persistent AKI (2)	AKD (3)	*P* value	In-between groups with *p* < 0.05(**)
	*n* = 655	*n* = 1009	*n* = 682		
Age (days) (M + IQR)	730 (120–2920)	1 (1–730)	1 (1–42)	< 0.001	(1) vs (2) and (1) vs (3)
Sex (%)					
Female	293 (44.7)	436 (43.2)	308 (45.2)	0.865	–
Environment (%)					
Urban	354 (54)	582 (57.7)	382 (56)	0.56	–
IH-AKI (%)	368 (56.2)	645 (63.9)	460 (67.4)	< 0.0001	–
AKI stages (%)					
1	233 (35.6)	208 (20.6)	67 (9.8)	< 0.0001	–
2	213 (32.5)	342 (33.9)	157 (23)		
3	209 (31.9)	459 (45.5)	458 (67.2)		
AKI causes					
Prerenal	570 (87)	858 (85)	515 (75.5)	< 0.0001	
Renal	73 (11.1)	133 (13.2)	154 (22.6)		–
Postrenal	12 (1.8)	18 (1.8)	13 (1.9)		
RRT (%)	2 (0.3)	4 (0.4)	19 (2.8)	< 0.0001	–
Pre-existing CKD (%)	24 (3.7)	28 (2.8)	36 (5.3)	0.113	–
Patients with following AKI episode (%)	50 (7.6)	82 (8.1)	52 (7.6)	0.9905	–
Number of following AKI episodes (A + SD)					
*	2.42 (0.67)	2.5 (0.97)	2.38 (0.63)	0.113	–
All patients	1.108 (0.419)	1.121 (0.494)	1.105 (0.406)	0.723	–
Baseline SCr (umol/l) (M + IQR)	27 (19–41.25)	25 (19–35)	32 (21–44)	< 0.001	(1) vs (2) and (2) vs (3)
Highest SCr (umol/l) (M + IQR)	72 (50–104)	77 (60–97)	113 (82–189)	< 0.001	(1) vs (3) and (2) vs (3)
Urea (mmol/l) (M + IQR)	5.45 (3.91–8.13)	7.03 (4.58–11.83)	16.09 (8.62–26.74)	< 0.001	(1) vs (3)
Uric acid (umol/l) (A + SD)	290.4 (161.61)	363.85 (236.49)	451.76 (307.16)	< 0.001	(1) vs (3) and (2) vs (3)
AST (U/l) (M + IQR)	35 (23–72)	47 (28–82)	57 (32–100.5)	< 0.001	(1) vs (2) and (3), (2) vs (3)
AAT (U/l) (M + IQR)	20 (13–43.75)	21 (11–52)	28 (12–79)	0.001	(3) vs (1) and (2)
Vs GGT (U/l) (A + SD)	118.56 (321.5)	132.63 (151.74)	230.33 (551.17)	0.005	(1) vs (3) and (2) vs (3)
Lactate dehydrogenase (U/l) (A + SD)v	758.81 (1484.01)	822.25 (1058.48)	1049.55 (1093.5)	< 0.001	(1) vs (3) and (2) vs (3)
Proteins (g/l) (A + SD)	54.14 (11.56)	49.84 (8.58)	47.27 (9.74)	< 0.001	(1) vs (2) and (1) vs (3)
Hemoglobin (g/dl) (A + SD)	10.7 (2.41)	10.63 (2.79)	9.43 (2.7)	< 0.001	(1) vs (3) and (2) vs (3)
Hematocrit (%) (A + SD)	31.59 (6.66)	30.81 (7.68)	27.24 (7.52)	< 0.001	(1) vs (3) and (2) vs (3)
Thrombocytes (n/mm3) (M + IQR)	244,000 (164,500–331,000)	213,000 (129,000–290,000)	147,500 (68,000–248,000)	< 0.001	(1) vs (3) and (2) and (2) vs (3)
Serum C-reactive protein (mg/l) (M + IQR)	10.23 (1.85–61.75)	12.26 (2.06–60.72)	19.08 (3.45–77.84)	0.002	(3) vs (1) and (2)
Procalcitonin (ng/ml) (A + SD)	2.74 (0.49–16.57)	4.75 (0.98–20.65)	5.99 (0.98–31.42)	< 0.001	(1) vs (2) and (3)
Ferritin (ng/ml) (A + SD)	77.5 (37–273)	215.5 (65–618)	295.5 (113–1144)	< 0.001	(1) vs (2) and (3), (2) vs (3)
Fibrinogen (mg/dl) (A + SD)	317.16 (168.24)	327.49 (165.26)	319.19 (168.22)	0.678	–
D-dimers (ng/ml) (A + SD)	900 (276–3491.75)	1138 (393–3503.25)	1862 (618–5889)	0.001	(3) vs (1) and (2)
Serum sodium (mmol/l) (A + SD)	137.58 (8.76)	135.16 (6.36)	134.24 (10.68)	< 0.001	(1) vs (2) and (1) vs (3)
Serum potassium (mmol/l) (A + SD)	4.7 (1.08)	4.9 (0.99)	4.84 (1.27)	0.022	(1) vs (2)

Legend: AKD = acute kidney disease; IH-AKI = in-hospital acquired AKI; CKD = chronic kidney disease; RRT = renal replacement therapy; *%* percentage, *A* average, *SD* standard deviation, *n* number, *y* years, *kg* kilograms, *cm* centimetres, *SCr* serum creatinine, *GGT* gamma-glutamyl transferase, *AST* aspartate aminotransferase, *ALT* alanine aminotransferase, *mmol* milimol, *umol* micromole, *U* units, *l* litre, *ng* nanograms

*Only the patients that had at least one subsequent AKI episode; Chi-square test for categorical variables; ANOVA test for continuous normally distributed variables or Kruskal-Wallis test for non-normally distributed ones

**Only for ANOVA tests;

In the transient AKI group, patients were older, had higher haemoglobin, haematocrit, platelet count, serum proteins and sodium, and lower gamma–glutamyl transferase (GGT), maximum SCr, urea and uric acid levels as compared to persistent AKI and acute kidney disease groups. Patients who progressed to acute kidney disease were younger (2.11 ± 4.78 years), with a higher degree of anaemia, with fewer platelets, higher procalcitonin, higher lactate dehydrogenase (LDH), higher GGT, higher urea and higher baseline and maximum SCr. There were no differences regarding baseline SCr levels between transient AKI and acute kidney disease, although both groups had higher baseline SCr levels compared to persistent AKI (*P* < 0.001), regardless of pre–existing CKD. There were no statistically significant differences between sex and demographics in the three groups. In-hospital-acquired AKI was present in more than half of the cases, with the highest incidence observed in the acute kidney disease group (67.4%). In the transient AKI group, there were no differences between AKI stages. In the persistent AKI group, almost half the patients presented stage three AKI (45.5%), and in the acute kidney disease group, more than two thirds were stage three AKI (67.2%). The pre-renal cause of AKI was the main cause of kidney injury regardless of AKI duration. The intrinsic cause of AKI increased (11.16% in transient AKI, 13.2% in persistent AKI and 22.6% in acute kidney disease) with longer kidney injury duration. Of the entire AKI cohort, 1.1% required renal replacement therapy, including 76% who were in the acute kidney disease group (19 out of 25 patients).

Pre–existing CKD was present in 3.8% of the children, 40.9% of whom developed acute kidney disease (36 out of 88 patients).

We evaluated the aetiology of AKI and the risk of developing transient AKI, persistent AKI and acute kidney disease, as shown in Supplemental Table 2.

**Table 2:** Multivariate logistic regression models for the risk of death in AKI subgroups

	Transient AKI		Persistent AKI		AKD		Total	
	OR (95%CI)	*P* value	OR (95%CI)	*P* value	OR (95%CI)	*P* value	OR (95%CI)	*P* value
Pre-renal AKI								
Hypovolemia/ dehydration	0.18 (0.06–0.51)	0.0013	–	–	–	–	–	–
Systemic vasodilatation	Baseline		Baseline		–	–	Baseline	
Hypoxia/ischemia	–	–	–	–	Baseline		–	–
Intrinsic AKI								
Renal microvasculature alteration	–	–	59.08 (3.97–878.42)	0.0031	–	–	3.57 (1.79–7.12)	0.0003
Acute tubular necrosis	–	–	46.75 (2.58–845.05)	0.0092	8.04 (2.35–27.48)	0.0009	10.46 (3.56–30.7)	< 0.0001
Acute tubulo-interstitial nephritis	–	–	–	–	0.18 (0.05–0.62)	0.01	0.31 (0.14–0.7)	0.0045
RRT	–	–	0.02 (0.00–0.7)	0.0316	–	–	0.39 (0.13–1.1)	0.0752
Proteins (g/l)	0.96 (0.93–0.99)	0.0435	0.94 (0.9–0.97)	0.0018	–	–	0.96 (0.95–0.98)	0.0017
Hemoglobin (g/dl)	0.87 (0.74–1.01)	0.0728	0.85 (0.75–0.96)	0.0121	0.83 (0.73–0.95)	0.0084	0.88 (0.82–0.95)	0.0011
Thrombocytes*10^3^ (mm3)	0.99 (0.98–0.99)	0.0002	0.99 (0.98–0.99)	< 0.0001	0.99 (0.98–0.99)	< 0.0001	0.99 (0.99–0.99)	< 0.0001
Serum C-reactive protein (mg/l)	0.99 (0.98–0.99)	0.0003	–	–	1.00 (1.00–1.00)	0.0272	–	–
Procalcitonin (ng/ml)	1.02 (1.008–1.032)	0.0008	–	–	–	–	1.00 (1.00–1.01)	0.005
AUC	0.852	< 0.0001	0.845	< 0.0001	0.838	< 0.0001	0.824	< 0.0001
Nagelkerke R square	0.421		0.325		0.345		0.304	

Regression model adjusted for age, sex, in-hospital acute kidney injury. Variables excluded from regressions: subsequent AKI episodes, pre-existing chronic kidney disease, intrinsic AKI—glomerulonephritis, and post-renal acute kidney injury

*AKI* acute kidney injury, *AKD* acute kidney disease, *RRT* renal replacement therapy, *mg* milligrams, *g* grams, *ng* nanograms, *mmol* milimols, *mm3* cube millimetre, *dl* decilitre, *l* litre, *OR* odds ratio, *CI* confidence interval, *AUC* area under the curve

Overall, pre-renal causes increased the risk of transient AKI 1.58-fold (95% CI 1.21–20.4, *P* = 0.0005), and intrinsic AKI increased the risk of acute kidney disease 2.11-fold (95% CI 1.67–2.65, *P* < 0.0001). Among the pre-renal causes of AKI, only hypovolemia increased the risk of transient AKI (OR = 10.69, *P* < 0.0001). Systemic vasodilatation and hypoxia/ischemia increased the risk of persistent AKI 1.71- and 1.56-fold, respectively (*P* < 0.0001). Among the pre-renal causes, only hypoxia/ischemia increased the risk of acute kidney disease (OR = 1.81, *P* < 0.0001). The intrinsic alterations that increased acute kidney disease included renal microvasculature alterations (OR = 1.94, *P* = 0.014), acute tubular necrosis (OR = 6.41, P < 0.0001), and acute tubulointerstitial nephritis (OR = 1.6, *P* = 0.0006).

**Fig. 1:**
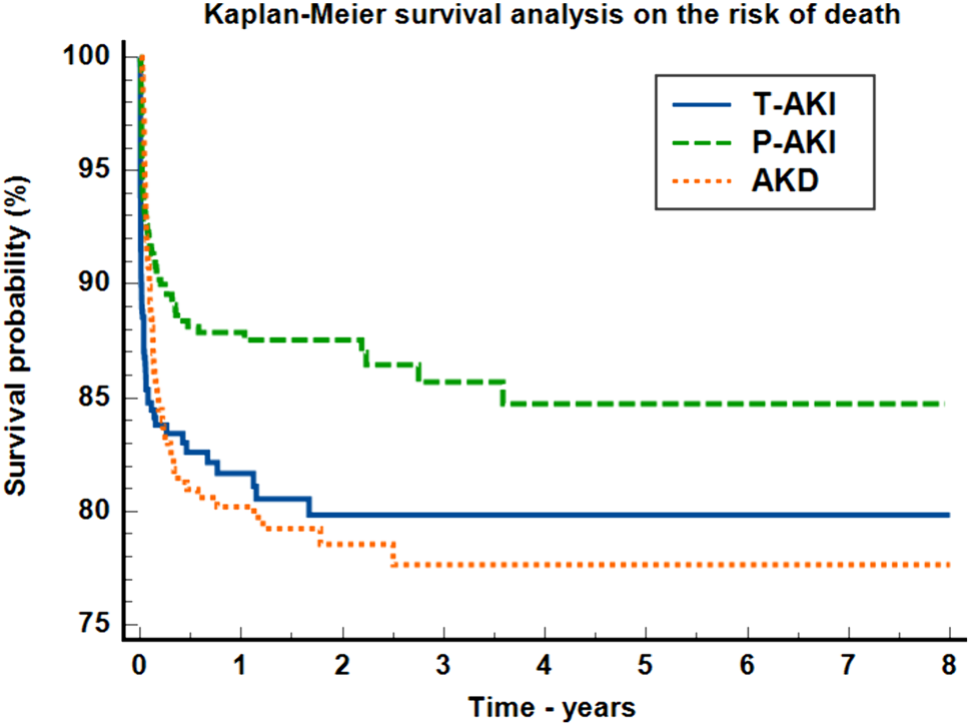
Kaplan–Meier survival analysis of the risk of death; *AKI* acute kidney injury, *T–AKI* transient AKI, *P–AKI* persistent AKI, *AKD* acute kidney disease

Overall AKI mortality was 13.1% (307 patients). Patients in the acute kidney disease group had the highest mortality rate, i.e., 16.42% (112 patients), followed by 14.66% in the transient AKI group (96 patients) and 9.81% in the persistent AKI group (99 patients). In order to evaluate the overall risk of death, we created a Kaplan–Meier survival curve, as shown in Fig. 1.

The number of censored cases differed among the three groups, with 85.34% for transient AKI, 90.19% for persistent AKI and 83.58% for acute kidney disease, respectively. The log rank test comparison of the curves was statistically relevant (*P* < 0.0001). The transient AKI (HR = 1.88, 95% CI 1.40–2.51) and acute kidney disease groups (HR = 1.47, 95% CI 1.13–1.90) presented a higher risk of death compared to the persistent AKI group. In the multivariable Cox proportional–hazards regression model, even after adjusting for age, sex, in–hospital-acquired AKI, AKI stage and AKI cause (Harrell’s C-index = 0.72, 95% CI 0.69–0.75), transient AKI presented a higher risk of death compared to persistent AKI (HR = 3.07, 95% CI 2.34–4.01, *P* < 0.0001).

We performed several logistic regression models in order to identify the mortality risk factors in each group. The variables were entered into the model using the backward method and proved to be good fit models, with area under the curve (AUC) higher than 0.82—Table 2.

In transient AKI, higher levels of procalcitonin and lower levels of serum proteins, haemoglobin, C-reactive protein and platelets increased the risk of death. In the persistent AKI group, the risk of death increased in the presence of renal microvascular alterations, acute tubular necrosis and lower serum levels of proteins, haemoglobin and platelets. In the acute kidney disease group, acute tubular necrosis, higher levels of C-reactive protein and reduced levels of haemoglobin and platelets increased the risk of death. In the regression model for overall mortality, renal microvasculature alterations, acute tubular necrosis, higher procalcitonin and lower levels of proteins, haemoglobin and platelets increased the risk of death.

**Fig. 2:**
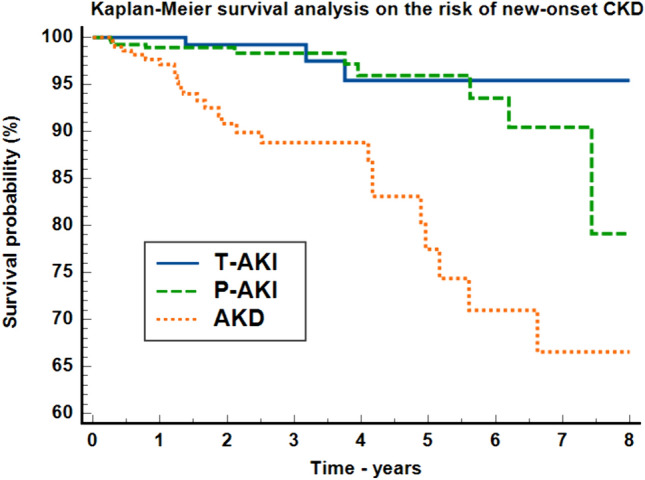
Kaplan–Meier survival analysis of new-onset CKD; *AKI* acute kidney injury, *T–AKI* transient AKI, *P–AKI* persistent AKI, *AKD* acute kidney disease

Patients undergoing renal replacement therapy had higher mortality rates than other patients did (OR: 4.54, 95% CI 2.02–10.2, *P* = 0.0002). Follow up lasted longer than 3 months for 962 out of 2346 patients, with a median follow–up of 17.53 months (IQR = 7.6–36.2 months). New-onset CKD occurred in 39 children (4.05%). CKD was influenced only by the intrinsic cause of AKI (OR = 4.69, 95% CI 2.49–8.85, P = 0.0021) and not by AKI severity. Kaplan–Meier survival curve analysis of CKD development is shown in Fig. 2.

There were 98.7% censored patients in the transient AKI group, 97.57% in the persistent AKI group and 91.87% in the acute kidney disease group, and the log–rank was statistically significant (*P* < 0.0001). The acute kidney disease group had a 7.42- (95% CI 3.15–17.45) and 4.07-fold (95% CI 1.93–8.57) higher risk of progressing to CKD as compared to the transient AKI and persistent AKI groups.

Multivariable Cox proportional–hazards regression model adjusted for age, sex, in–hospital-acquired AKI, AKI causes and subsequent AKI episodes (Harrell’s C-index = 0.76, 95% CI 0.66–0.86), maintained acute kidney disease as an independent factor for CKD development (HR = 5.35, 95% CI 2.67–10.71, *P* < 0.0001). Patients with prior CKD had no significant risk of recurrent AKI (OR = 1.18, 95% CI 0.56–2.48, *P* = 0.443).

## Discussion

In this study, we compared the different outcomes according to AKI duration in children. We identified the temporal evolution of AKI based on kidney injury duration. The discrimination between the biological parameters, AKI causes and AKI severity was significant among the three groups of transient AKI,  persistent AKI, and acute kidney disease. Patients with acute kidney disease presented the highest mortality rates, followed by those in the transient AKI and persistent AKI groups, respectively. The underlying disease severity, reflected by the biological parameters, as well as disease evolution and management are associated with these outcomes. Besides younger age, the biological parameters from day one of AKI and the intrinsic cause were associated with a prolonged AKI episode. Regardless of AKI severity, progression to CKD was higher in patients with acute kidney disease and in children with intrinsic AKI. Subsequent AKI episodes did not increase the risk of CKD.

A recent meta–analysis of AKI incidence in hospitalised children reported a high incidence of AKI (26%) [16]. However, we report a higher incidence than the 0.39% AKI incidence reported by Sutherland in an American population [17], probably because we incorporated a wider spectrum of ages (including neonates). Indeed, we divided the cohort based on the duration of AKI and we found two studies that followed this division [10, 11].

In the first one, Nagata et al., compared the effects of transient AKI, persistent AKI and acute kidney disease on the long–term renal prognosis in adult settings [11]. In their study, persistent AKI and acute kidney disease had higher incidences (40%) compared to transient AKI (19%) [11]. Conversely, LoBasso et al., found transient AKI in 87% of the children undergoing cardio-pulmonary by-pass who developed AKI, followed by 6.7% in persistent AKI, and 6.2% in acute kidney disease, respectively [10]. Our results are closer to those reported in adults, despite the larger number of transient AKI cases in our cohort. The effect of kidney injury duration depends on the initiating AKI event, renal functional reserve and proper management. This is why, in the latter study, the initiating event, represented by cardio-pulmonary by-pass, induced a pre-renal AKI episode that resolved within 48 h [10]. Unlike Lo Basso’s cohort, our study included patients from both intensive care units and hospitalisations, from all medical specialities, including neonates. The reported incidence of acute kidney disease in children is heterogeneous, ranging between 6.3% [10] and 56.3% [19], and information is scarce [7, 9, 10, 18, 19]. The discrepancy regarding AKD incidence comes from smaller, retrospective observational studies that analysed specific groups at risk for AKI. For instance, Patel included 528 children with AKI from all paediatric units, yet he reported the highest incidence of acute kidney disease (56.3%). However, he found a much lower acute kidney disease incidence in patients undergoing non-kidney organ transplantation (13%). Similarly, the 35.3% acute kidney disease incidence reported by Daraskevicius in children who underwent allogeneic haematopoietic stem cell transplantation seems to be overestimated due to the reduced number of cases (51) [18]. Yet, in some instances, AKI evolution is strongly influenced by the initial AKI episode management, thus resulting in a reduced number of patients progressing to acute kidney disease [10]. Our results (29.1%) are comparable to a previous study that included patients aged from 1 month to 18 years old, with an overall incidence of acute kidney disease of 42.3% [9].

Pre-renal causes were the leading ones in all three groups. Decreased renal perfusion was the main risk factor for developing transient AKI. Rapid reversal of AKI was dependent on its cause. Unlike adults, children have a higher renal reserve in the absence of chronic diseases (i.e.: diabetes mellitus, arterial hypertension, cardiovascular disease). In the absence of early recovery, moderate to severe AKI incidence increases, mirroring the severity of the underlying disease. Prolonged pre-renal injury can lead to intrinsic damage because of decreased renal perfusion, hypoxia and high levels of pro–inflammatory cytokines [20]. In our persistent AKI group, the presence of an intrinsic cause of AKI increased the risk of progressing to acute kidney disease. In this group the incidence of inflammatory conditions (sepsis, septic shock, systemic inflammatory response syndrome, and hypoxia) is higher and reversal takes longer as compared to hypovolemic states. Our results are consistent with previous studies showing that, regardless of AKI duration or AKI severity, children with AKI have poor outcomes [7, 8].

Similar to published data, our study also identified biochemical risk factors associated with progression of AKI to acute kidney disease, these include lower haemoglobin, lower platelet counts, lower serum proteins and higher inflammatory markers as well as higher urea and higher baseline and maximum SCr levels [18, 19]. While transient AKI is considered a “functional” AKI with rapid reversal, persistent kidney injury over time may generate renal remodelling (i.e. tubulointerstitial inflammation and fibrosis) [20]. Children with prolonged AKI are often exposed to nephrotoxic medication that further contributes to the continuum of renal injury [20]. Nevertheless, progression to acute kidney disease is multifactorial. In acute kidney disease, there is an obvious shift from pre-renal towards intrinsic damage and intrinsic causes of AKI modulate AKI duration [20].

In this retrospective cohort study, we observed that patients who progress from AKI to acute kidney disease are at high risk of developing CKD. Several studies involving adults underline the high risk of progression to CKD after acute kidney disease, ranging from 14.7% to 37.4% [21–24]. Our results showed a lower incidence of new-onset CKD following an AKI episode (4.05%) when compared to Patel (8%-19%) [7, 19]. Nevertheless, patients in the acute kidney disease group had a fivefold increased risk of progressing to CKD, similar to results from the most extensive meta–analysis in adults [24] and to Patel’s results [19]. Interestingly, we found a high incidence of acute kidney disease in patients with pre-existing CKD (40.9%). Thus, CKD increases the susceptibility to acute kidney disease. We also highlighted that subsequent AKI episodes did not influence the risk of new-onset CKD. We did not evaluate the impact of other risk factors such as number of days of nephrotoxic medications, post-AKI proteinuria or chronic underlying diseases (even though in paediatric populations the incidence of chronic diseases is very low).

Currently, data regarding CKD development in paediatric AKI survivors suggest that certain conditions confer a greater risk for new-onset CKD. For instance, the Translational Research Investigating Biomarker Endpoints in AKI (TRIBE-AKI) study showed that perioperative AKI did not increase the risk of CKD 5 years after paediatric cardiac surgery [25]. In addition, the Follow-Up Renal Assessment of Injury Long-Term After Acute Kidney Injury (FRAIL-AKI) study did not identify cardiopulmonary bypass AKI as a risk factor of progression to CKD even though several novel urinary biomarkers remained elevated up to 7 years after an AKI episode [26]. On the other hand, Madsen linked perioperative AKI with a 3.8-fold higher risk of CKD in patients developing AKI after cardiac surgery over a follow-up of almost 5 years [27]. It is not possible to affirm that AKI causes CKD in children, yet certain factors seem to increase the risk. Unlike adults, where various AKI-associated factors can increase the risk of CKD (such as AKI severity, number of AKI episodes) [28], in our paediatric cohort only acute kidney disease and intrinsic AKI cause increased the risk of CKD. Similar results were published by Patel [7, 19].

Both AKI and acute kidney disease were associated with mortality among hospitalised patients [9, 15, 17]. AKI mortality was previously reported by Meena to be around 11%, similar to our results (13.1%) [16]. The reported acute kidney disease mortality in paediatric patients ranges from 10% to 31.8% [9, 10, 18]. Mortality increases with severity of kidney injury. Similar to previous studies, acute kidney disease shows the highest mortality rates [9–11, 18, 19]. Deng reported a 10.2% mortality rate in patients with acute kidney disease [8], similar to our results, yet much lower than the 37.7% and the 31.8% mortality rates reported by Patel and LoBasso, respectively [10, 19]. However, when compared to the literature, our transient AKI group showed unexpectedly high mortality rates, 14.66% compared to the 8.9% rate reported mortality by Patel [10]. Although transient AKI can be rapidly reversed, the associated hemodynamic changes observed in the acute state of pre-renal AKI seem to increase the mortality risk along with other associated comorbidities. In addition, our cohort comprised a mixed paediatric population, including neonates. However, it is fair to assume that some patients may have been included in the transient AKI group at the time, but they may have actually had a prolonged AKI evolution before admission.

This study aimed to assess if AKI–acute kidney disease–CKD is a continuum of time-dependent renal injury associated with high morbidity and mortality. In summary, we found that new onset CKD was not influenced by AKI severity or subsequent AKI episodes but by AKI cause and duration. Acute kidney disease is an independent risk factor for CKD. While rapidly resolving AKI has worse survival rates compared to patients with prolonged AKI, progression to CKD is lower. The high mortality rates prove that these patients do not die of AKI but rather, with AKI complicating the course of the underlying disease.

The main limitation of our study is the single centre and retrospective nature. In addition, the lack of urine output information is a major limitation in AKI diagnosing and staging. Another limitation is represented by the lack of data on exposure to nephrotoxins, post-AKI proteinuria, chronic diseases etc., to be considered in the analysis of the risk of developing new-onset CKD. In addition, the lack of information on paediatric intensive care unit (PICU) admission and/or length of stay may limit the strength of our results. The absence of baseline SCr in patients with contrast-associated-AKI increases the difficulty of correctly staging AKI. Due to the retrospective nature of the study, it is possible that some of the patients in the transient AKI group should have been assigned to the persistent AKI or acute kidney disease groups at inclusion. Future prospective studies need to address this issue and shed more light on the impact of AKI duration on mortality in the paediatric population. The strengths of our study include the large number of patients from all paediatric age groups, allowing to have new insights on AKI duration, and acute kidney disease development and progression to CKD following an AKI episode. Prospective studies with standardised follow-up protocols are needed in the paediatric field in order to validate our findings and overcome their limitations.

This study underlines that the continuum of kidney injury should be addressed by including the duration of AKI along with the function-based stages and quasi-anatomic nomenclature. Physicians should reconsider the cause and disease management plans when kidney injury lasts longer than 48 h.

## Supplementary Information

Below is the link to the electronic supplementary material.

Supplementary file1 (DOCX 12 KB)

Supplementary file2 (DOCX 16 KB)

Supplementary file3 (JPG 32 KB)

## Data Availability

The data collected for this study will be available for others, upon request directly to the corresponding author. Data will be available as deidentified participant data. Informedconsent forms and statistical analysis plan will be available upon request. The data will be available with publication. The data will be available upon request at the e–mail address mgafencu@umft.ro, and will be shared after direct request to and approval of the proposal by all the authors.
