# Supplementary material for: Outcomes of acute kidney injury continuum in children
Source: J Nephrol. 2024 Oct 24;37(9):2569–78. doi: 10.1007/s40620-024-02097-1 (PMC11663817; doi:10.1007/s40620-024-02097-1)
Supplement: Supplementary file 1 — Supplementary file1 (DOCX 12 KB) [file 40620_2024_2097_MOESM1_ESM.docx]

Supplemental table 1 – AKI and neonatal AKI KDIGO criteria using serum creatinine values

| Stage | AKI in children older than 1 month | Neonatal AKI |
| --- | --- | --- |
| 1 | Increase in serum creatinine of ≥0.3 mg/dl or increase 1.5-1.9 times from baseline | Increase in serum creatinine of ≥0.3 mg/dl or increase 1.5-1.9 times from baseline |
| 2 | Increase in serum creatinine 2-2.9 times from baseline | Increase in serum creatinine 2-2.9 times from baseline |
| 3 | Increase in serum creatinine 3 times from baseline or increase in serum creatinine ≥4mg/dl or initiation of renal replacement therapy or decrease in eGFR<35ml/min/1.73sm in patients < 18 years | Increase in serum creatinine 3 times from baseline or increase in serum creatinine ≥2.5mg/dl or initiation of renal replacement therapy |

Legend: AKI=acute kidney injury, eGFR=estimated glomerular filtration rate, ml=millilitres, kg=kilogram, h=hour, mg=milligram, dl=decilitre.
