# Supplementary material for: Outcomes of acute kidney injury continuum in children
Source: J Nephrol. 2024 Oct 24;37(9):2569–78. doi: 10.1007/s40620-024-02097-1 (PMC11663817; doi:10.1007/s40620-024-02097-1)
Supplement: Supplementary file 2 — Supplementary file2 (DOCX 16 KB) [file 40620_2024_2097_MOESM2_ESM.docx]

**Supplemental table 2. AKI causes on aetiology and the risk of developing AKI in time: transient, persistent and acute kidney disease**

| AKI cause |  | Transient AKI | | Persistent AKI | | AKD | |
| --- | --- | --- | --- | --- | --- | --- | --- |
|  |  | OR (95% CI) | *P* value | OR (95% CI) | *P* value | OR (95% CI) | *P* value |
| Prerenal | *Hypovolemia/ Dehydration* | 10.69 (8.63-13.25) | <0.0001 | 0.38 (0.31-0.47) | <0.0001 | 0.14 (0.1-0.19) | <0.0001 |
|  | *Systemic vasodilatation* | 0.38 (0.3-0.48) | <0.0001 | 1.71 (1.43-2.05) | <0.0001 | 1.18 (0.97-1.43) | 0.086 |
|  | *Hypoxia/ Ischemia* | 0.23 (0.17-0.3) | <0.0001 | 1.56 (1.3-1.86) | <0.0001 | 1.81 (1.49-2.19) | <0.0001 |
| Renal | *Renal microvasculature alterations* | 1.52 (0.88-2.62) | 0.13 | 0.31 (0.15-0.6) | 0.0005 | 1.94 (1.14-3.3) | 0.014 |
|  | *Glomerulonephritis* | 0.12 (0.01-0.95) | 0.0449 | 1.2 (0.51-2.85) | 0.6687 | 2.23 (0.94-5.29) | 0.067 |
|  | *Acute tubular necrosis* | 0.22 (0.05-0.94) | 0.0417 | 0.32 (0.12-0.87) | 0.0263 | 6.41 (2.66-15.43) | <0.0001 |
|  | *Acute tubule-interstitial nephritis* | 0.58 (0.42-0.81) | 0.0015 | 0.96 (0.74-1.26) | 0.8074 | 1.6 (1.22-2.1) | 0.0006 |
| Postrenal | *Urinary tract malformation/ obstruction* | 1.06 (0.54-2.1) | 0.8461 | 0.84 (0.44-1.59) | 0.603 | 1.13 (0.58-2.2) | 0.707 |

Legend: AKI=acute kidney injury; OR=odds ratio; CI=confidence interval; Systemic vasodilatation: sepsis, septic shock, systemic inflammatory response syndrome; Renal microvasculature alterations: typical and atypical haemolytic uremic syndrome, intravascular disseminated coagulation.
