# Supplementary figures and images for: Outcomes of acute kidney injury continuum in children
Source: J Nephrol. 2024 Oct 24;37(9):2569–78. doi: 10.1007/s40620-024-02097-1 (PMC11663817; doi:10.1007/s40620-024-02097-1)

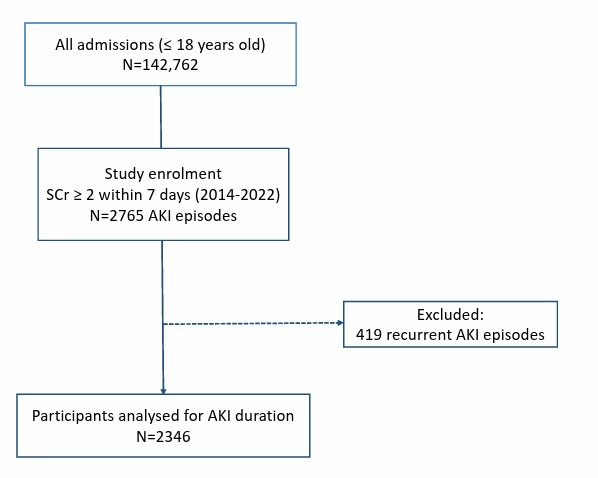

Supplement: Supplementary file 3 — Supplementary file3 (JPG 32 KB) [file 40620_2024_2097_MOESM3_ESM.jpg]
